# Variations in leaf phyllosphere microbial communities and development of tobacco brown spot before and after fungicide application

**DOI:** 10.3389/fmicb.2022.1068158

**Published:** 2022-11-17

**Authors:** Li-Gang Xiang, Han-Cheng Wang, Liu-Ti Cai, Tao Guo, Fei Luo, Tom Hsiang, Zhi-He Yu

**Affiliations:** ^1^College of Agriculture, Yangtze University, Jingzhou, Hubei, China; ^2^Guizhou Provincial Academician Workstation of Microbiology and Health, Guizhou Academy of Tobacco Science, Guiyang, Guizhou, China; ^3^College of Life Sciences, Yangtze University, Jingzhou, Hubei, China; ^4^School of Environmental Sciences, University of Guelph, Guelph, ON, Canada

**Keywords:** microbial community, STROBY, tobacco brown spot, leaf phyllosphere, high-throughput sequencing

## Abstract

In recent years, STROBY (50% Kresoxim-methyl) has been widely used to control tobacco brown spot in Guizhou Province, China. As a broad-spectrum fungicide, STROBY targets not only phytopathogens, but also affects many other microorganisms including those pathogenic, beneficial, or neutral to the plant hosts. To understand the effects of STROBY on the phyllosphere microbial communities of tobacco leaves during the development of tobacco brown spot, the fungal and bacterial communities of symptomatic and asymptomatic leaves at four time points, before spraying (August 29) and after spraying (September 3, 8, and 13), were investigated using the Illumina high-throughput sequencing. The results showed that STROBY had significant effects on the phyllosphere microbial communities of tobacco leaves. Microbial communities in asymptomatic leaves were more greatly affected than their counterparts in symptomatic leaves, and fungal communities were more sensitive than bacterial communities. Throughout the experiment, the most common genera in symptomatic leaves were *Alternaria*, *Pseudomonas*, *Pantoea*, and *Sphingomonas*, and in asymptomatic leaves, these were *Golubevia* and *Pantoea*. After spraying, the alpha diversity of fungal communities increased in symptomatic leaves and decreased in asymptomatic leaves, while the alpha diversity of bacteria increased in both types of leaves. Beta diversity showed that in asymptomatic leaves, the fungal communities in the first stage was significantly different from the remaining three stages. In contrast, the fungal communities in symptomatic leaves and the bacterial communities in all leaves did not fluctuate significantly during the four stages. Before spraying (August 29), the dominant functions of the fungal community were animal pathogen, endophyte, plant pathogen, and wood saprotroph. Whereas after spraying (September 3, 8, and 13), the proportion of the above fungal functions decreased and the unassigned functions increased, especially in asymptomatic leaves. This study describes the effects of STROBY application and tobacco brown spot presence in shaping the leaf phyllosphere microbial communities, and provides insights into the microbial community effects on tobacco leaves of a strobilurin fungicide.

## Introduction

Tobacco brown spot caused by *Alternaria alternata* (Fr.) Keissl, has been confirmed as one of the most harmful fungal diseases that significantly affects the quantity and quality of tobacco (Wang et al., 2015). In China, approximately 100,000 ha of tobacco fields are affected by tobacco brown spot each year, resulting in an economic loss of 1 billion RMB (Liu et al., 2009). Symptoms of tobacco brown spots on leaves include small, round, and yellowish-brown spots in the initial stage, which then gradually develop into large areas (Jing et al., 2018). The most effective and economical method of controlling this disease in the field is the application of fungicides. Fungicides that have been used for the control of tobacco brown spot in China are boscalid (Lei et al., 2021), azoxystrobin, and difenoconazole (Wang et al., 2016). In recent years, a broad-spectrum fungicide, STROBY (50% kresoxim-methyl), has been widely used in Guizhou Province for tobacco spot disease control, especially for tobacco brown spot. STROBY is a Quinone outside Inhibitor (QoI) fungicide (Kolosova et al., 2017; Ge et al., 2021), also commonly called strobilurins. Strobilurins bind to the Qo site of cytochrome b, blocking electron transfer between cytochrome b and cytochrome c1 and inhibiting mitochondrial respiration (Balba, 2007; Mercader et al., 2008). Strobilurins are known to affect Ascomycota, Basidiomycota, and even Oomycota (Grossmann and Retzlaff, 1997), but there are few reports on whether it has an effect on bacteria. In previous studies, STROBY was used to control Botrytis blight of gladiolus (Singh et al., 2011), and powdery mildew of mango, cucumber, and grape (Yu et al., 2008; Reuveni et al., 2018). In addition to its use as a fungicide, STROBY can also be used as a growth-promoting compound to regulate leaf growth in *Arabidopsis* (Van Dingenen et al., 2017) and to promote wound healing in potato tubers (Ge et al., 2021).

The phyllosphere is the aerial or above-ground part of plant, such as stems, leaves, flowers, and fruits (Knief et al., 2010). From a microbial point of view, the environmental conditions of the leaf phyllosphere may be harsh, with limitations on free water, nutrients, favorable temperatures, and relative humidity, as well as fluctuations in UV radiation (Bashir et al., 2022). Anthropogenic factors such as climate warming, chemical and air pollution, and application of fertilizers and pesticides have wide impacts on leaf phyllosphere microorganisms (Xu et al., 2022). Because of the quickly changing environmental conditions of the phyllosphere, the microbial communities are also in flux. Within these communities, some members may be beneficial to the host plant, and improve plant productivity, or defend against phytopathogen invasion, or increase plant stress resistance, while others are plant pathogens that can cause disease once conditions are suitable, but most microorganisms are likely neutral and have no effects on the host (Stone et al., 2018; Thapa and Prasanna, 2018; Liu et al., 2020).

As a broad-spectrum fungicide, STROBY affects not only plant pathogens but also microorganisms that are beneficial or neutral to hosts. Therefore, the objectives of this study were as follows: (i) assess field efficacy of STROBY against tobacco brown spot; (ii) investigate composition and diversity of microbial communities in the leaf phyllosphere before and after STROBY application; and (iii) compare differences in microbial community composition and diversity and response to STROBY pressure between symptomatic and asymptomatic leaves.

## Materials and methods

### Experimental design

The experiment was conducted in August and September 2020 in a mature tobacco field, Bijie city (26.74 N, 104.02E), Guizhou Province, China. Prior to the experiment, tobacco brown spot had sporadically appeared on the tobacco plants (cultivar Yunyan 105) in the field. On 29 August 2020, nine symptomatic and nine asymptomatic tobacco plants at least 3 m apart from each other were randomly selected for first sampling, and one tobacco leaf was collected from each plant. After the first sampling, the entire tobacco field (50 m × 20 m) was sprayed with a 3,000-fold dilution (300 g/ha) of STROBY® (50% Kresoxim-methyl, BASF), and the tobacco leaves were sprayed on both sides until runoff. The second, third, and fourth samplings were done on September 3, 8, and 13, respectively. The 18 tobacco plants used for sampling were the same ones at all four sampling time points. The letters CBB were used to denote symptomatic leaves and CBJ to denote asymptomatic leaves, followed by the number 0 for the first sampling, 1 for the second sampling, 2 for the third sampling, and 3 for the fourth sampling, and finally followed by 1–3 for biological repetition.

During the entire experiment, environmental conditions were measured with an automatic weather station (Beijing Xinhong Tec. IT, Co., Ltd., Beijing, China) and soil moisture sensor (RainPoint, Walnut, CA, United States). Temperature, relative humidity, rainfall, soil temperature, soil relative humidity, and disease index were the average of the 4 days before sampling and the day of sampling. The calculation method of disease index followed Wu et al. (2015), and the classification of tobacco brown spot followed the Chinese National Standard (GB/T 23222-2008) from the Standardization Administration of China.

Leaf phyllosphere microorganisms were collected as described by Luo et al. (2019), with slight modifications. Three leaves of the same type and time point were mixed for processing giving three samples of symptomatic leaves and three samples of asymptomatic leaves. Each leaf was cut into 5 mm by 5 mm pieces. Five grams of leaf pieces per sample were placed in a 500 ml sterile conical flask with 300 ml phosphate buffer saline (PBS) containing 0.01% Tween-80. Then the flask was shaken at 28°C for 30 min at 180 rpm/min. The PBS buffer was then filtered through a 0.45 μm filter microfiltration membrane, and the membrane was stored at-80°C for further DNA extraction.

### DNA extraction, PCR amplification, and high-throughput sequencing

Total genomic DNA from each of the 24 samples was extracted using a FastDNA® Spin Kit (MP Biomedicals, Santa Ana, CA, United States) based on the manufacturer’s instruction. The purity and concentration of genomic DNA were assessed using agarose gel electrophoresis and the NanoDrop-2000 (Thermo Fisher Scientific, Waltham, MA, United States). The DNA concentration was diluted to 30 ng/μl for each sample. For PCR amplification, the bacterial hypervariable V4 region of the 16S rRNA gene was amplified using primers 515F/806R (Moreau and Rubin, 2017), and the fungal ITS1 region was amplified using primers ITS1/ITS2 (Zhang et al., 2010). The PCR reaction solutions and thermocycling steps for V4 and ITS1 regions followed Wei et al. (2018). The PCR products were visualized on 2% agarose gel electrophoresis, quantified by NanoDrop-2000, and purified with the Qiagen Gel Extraction Kit (Qiagen, Dusseldorf, Germany). Libraries were generated for each sample as described in Xiang et al. (2022). Finally, the libraries were sequenced on an Illumina NovaSeq PE250 platform at Novogene Bioinformatics Technology Co., Tianjin, China, generating 250 bp paired-end reads.

### Bioinformatics analysis

Paired-end raw reads were assigned to samples based on their unique 12-bp barcodes and truncated by cutting off the barcode and primer sequence. Paired-end reads with at least 10 bp overlap and up to 0.25 mismatch rate were merged using FLASH V1.2.7 (http://ccb.jhu.edu/software/FLASH/; Magoč and Salzberg, 2011). QIIME V1.9.1^1^ was used to filter raw reads under specific criteria (reads with ambiguous bases or quality scores <20 or the length < 200 bp) to obtain clean reads (Bokulich et al., 2013). Clean reads were compared to the UNITE (V12.01.2017) and Silva (V132) databases using the UCHIME algorithm^2^ to detect chimeric sequences for removal (Haas et al., 2011). After a series of operations, the sequences were considered “effective” reads. Sequences with at least 97% similarity were assigned to the same operational taxonomic unit (OTU) using Uparse software V7.0.1001 (Edgar, 2013). Based on the Mothur algorithm, the taxonomic information of each representative sequence of each OTU was annotated using the UNITE and Silva database. To analyze the alpha and beta diversity of the microbial communities for each sample, the OTU abundance was normalized to the sample with the fewest sequences. All alpha indices of microbial communities in the samples were calculated with QIIME V1.7.0, and displayed with R software V2.15.3. The beta diversity of both weighted and unweighted UniFrac distance was calculated with QIIME V1.9.1. The Wilcoxon signed rank test was used to compare the alpha diversity index of symptomatic and asymptomatic leaf microbial communities. Principal co-ordinate analysis (PCoA) with unweighted UniFrac distance was performed based on the Bray–Curtis dissimilarity matrix, and the results were displayed using the WGCNA package, stat packages, and ggplot2 package in R software following Zhou et al. (2018). Linear discriminant analysis effect size (LEfSe) analysis with a threshold of 4.0 in the logarithmic LDA score was used to reveal the significant ranking of biomarkers between samples. Effective reads were compared with the Greengene database using PICRUSt^3^ to predict bacterial functionality (Langille et al., 2013), and with FUNGuild database^4^ to obtain the fungal trophic mode and guild (Nguyen et al., 2016). The co-occurrence networks of leaf phyllosphere microbial genera and the relationships between environmental factors and microbial genera richness were demonstrated using Spearman’s rank analysis based on significant (*p* < 0.05) and strong positive correlations (*r* > 0.6) or strong negative correlations (*r* < −0.6; Li and Wu, 2018).

## Results

### Environmental conditions and tobacco disease index

During the 5-day period of each sampling interval (August 25–29, August 30 to September 3, September 4–8, and September 9–13), there were no significant differences in average daily temperatures, rainfall, and soil temperature between the four stages; the average daily relative humidity in the first stage was significantly lower than that in the fourth stage; and the average daily soil relative humidity and disease index in the first and second stages were significantly lower than that in the third and fourth stages (Table 1). As asymptomatic plants did not develop disease throughout the experiment, only the disease index of symptomatic plants was counted.

**Table 1:** Environmental factors and symptomatic tobacco plant disease indices in tobacco fields.

Sampling date	Temperature (°C)	Relative humidity (%)	Rainfall (mm/m^2^)	Soil temperature (°C)	Soil relative humidity (%)	Disease index
Aug 25	19.0	62.2	0.0	21.7	27.3	17.9
Aug 26	17.6	65.5	0.0	21.1	24.9	18.0
Aug 27	22.2	62.7	0.0	21.7	22.6	18.0
Aug 28	19.2	82.4	1.4	21.6	21.5	18.2
Aug 29	18.3	82.5	13.6	21.1	23.5	18.3
Average	19.3 ± 1.8a	71.1 ± 10.5b	3.0 ± 6.0a	21.4 ± 0.3a	24.0 ± 2.2b	18.1 ± 0.2c
Aug 30	19.1	80.4	0.6	21.2	25.2	20.0
Aug 31	19.8	77.8	3.0	21.3	24.8	20.0
Sep 1	19.6	73.4	0.0	21.5	23.7	20.6
Sep 2	19.9	77.3	0.0	21.5	22.3	20.6
Sep 3	19.0	83.8	19.0	21.2	27.7	20.8
Average	19.5 ± 0.4a	78.5 ± 3.9ab	4.5 ± 8.2a	21.3 ± 0.2a	24.7 ± 2.0b	20.4 ± 0.4c
Sep 4	19.8	81.2	1.2	21.5	30.2	21.6
Sep 5	20.2	81.9	2.2	21.8	28.6	23.2
Sep 6	16.1	89.5	53.6	20.1	33.2	25.7
Sep 7	17.5	74.8	0.0	21.0	32.1	27.4
Sep 8	17.5	79.0	0.0	21.4	31.1	28.3
Average	18.2 ± 1.7a	81.3 ± 5.4ab	11.4 ± 23.6a	21.2 ± 0.7a	31.0 ± 1.8a	25.2 ± 2.8b
Sep 9	18.9	79.1	0.0	21.9	30.6	28.5
Sep 10	17.4	89.8	11.8	20.8	32.5	29.0
Sep 11	16.1	86.5	0.0	20.2	32.5	30.0
Sep 12	16.8	90.8	13.0	20.2	32.9	31.6
Sep 13	16.5	91.0	25.4	19.9	34.4	32.2
Average	17.1 ± 1.1a	87.4 ± 5.0a	10.0 ± 10.6a	20.6 ± 0.8a	32.6 ± 1.4a	30.3 ± 1.6a

Different letters in the same column indicate significant difference at *p* < 0.05 among stage.

### Sequence information

After sequence and data processing, a total of 794,733 and 790,491 effective fungal reads were obtained from the symptomatic and asymptomatic leaves, respectively, averaged across three samples each (Supplementary Table S1). The average number of reads in symptomatic and asymptomatic leaves at four sampling time points was 64,000–68,000. For bacterial communities, 833,289 effective reads were obtained from symptomatic leaves, and the average number at four sampling time points ranged from 62,000 to 85,000. The asymptomatic leaves gave 835,795 effective bacterial reads with averages ranging from 66,000 to 78,000 among the four sampling time points. The fungal and bacterial raw sequences of each sample have been deposited in the SRA database under project number PRJNA806570.

### Microbial community composition

**Figure 1:**
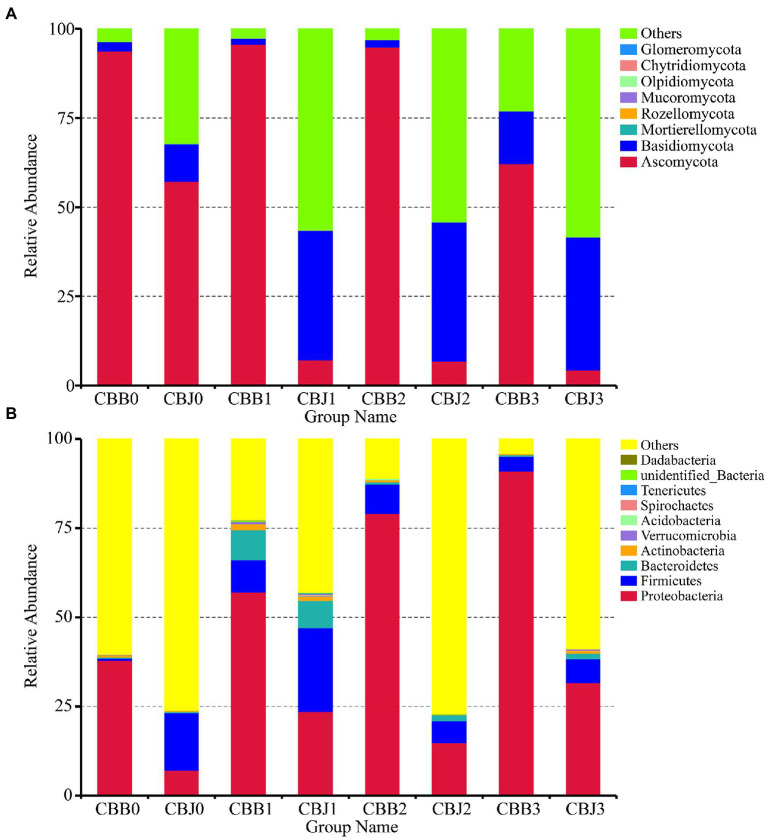
Microbial community composition of symptomatic (CBB) and asymptomatic (CBJ) tobacco leaves at four sampling time points (0–3) at the phylum levels (**A** for fungi, **B** for bacteria). The phyla with relative abundance less than 0.01% or without identification were classified as “Others.”

**Figure 2:**
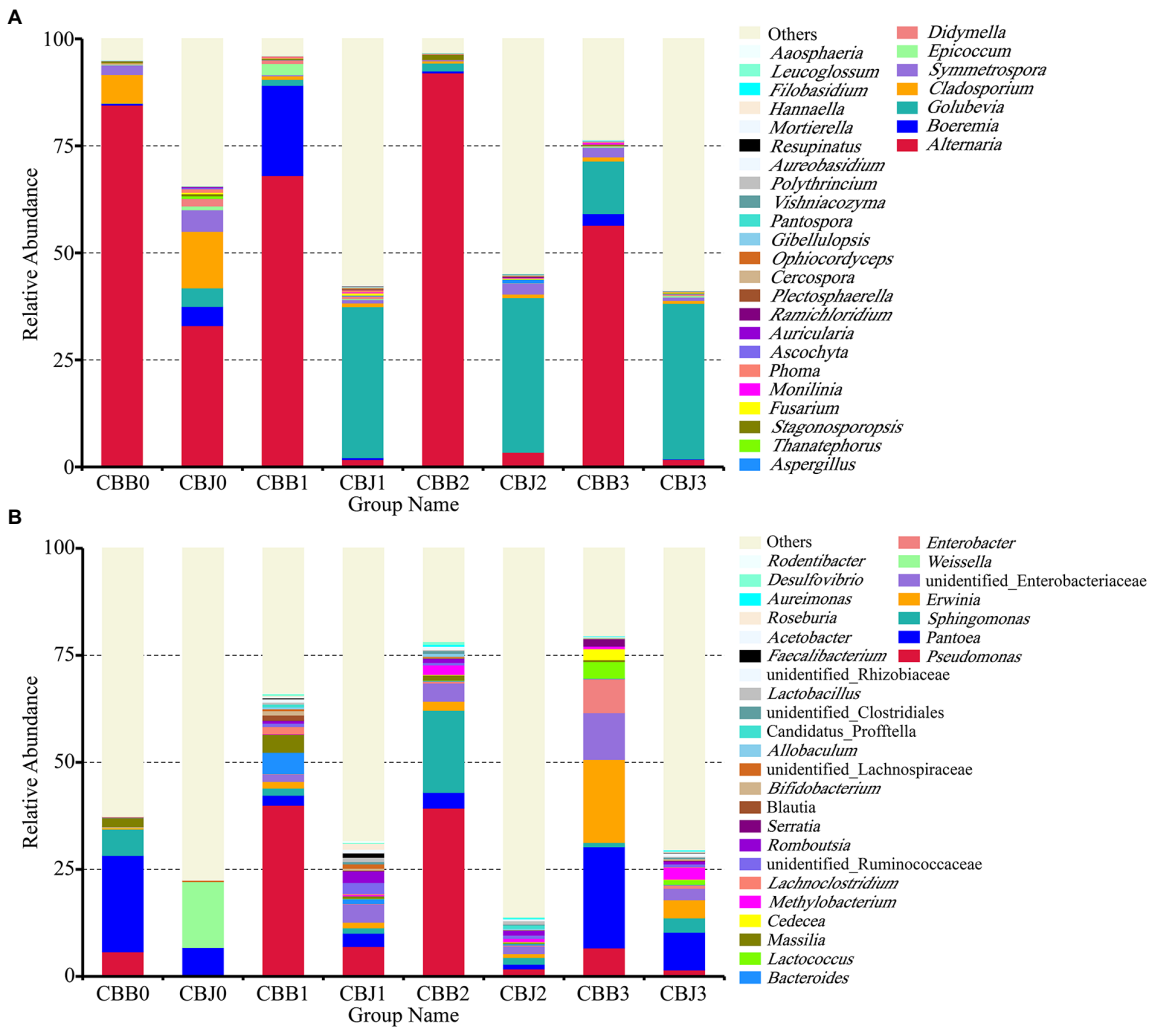
Microbial community composition of symptomatic (CBB) and asymptomatic (CBJ) tobacco leaves at four sampling time points (0–3) at the genus leaves (**A** for fungi, **B** for bacteria). The genera with relative abundance less than 0.01% or without identification were classified as “Others”.

At the phylum level, the fungal divisions Ascomycota and Basidiomycota were the most abundant in symptomatic and asymptomatic leaves, except for the “Others” group (Figure 1A). The “Others” group in Figures 1, 2 represent all microorganisms that were unidentified or whose relative abundance was less than 0.1%. In symptomatic leaves, the relative abundance of Ascomycota remained at about 94% for 10 days after spraying, while it decreased significantly to 62% on the 15th day after spraying. In asymptomatic leaves, Ascomycota was the dominant fungal division (57%) followed by Basidiomycota (10%) before spraying. On the fifth day after spraying, the relative abundance of Ascomycota decreased significantly to 7%, while the relative abundance of Basidiomycota increased significantly to 36% and became the most dominant phylum. The relative abundance of Proteobacteria gradually increased after spraying, with 38/7, 57/24, 79/15, and 91/32% for symptomatic/asymptomatic leaves at the four sampling time points, respectively (Figure 1B).

At the genus level, *Alternaria* was the dominant genus in symptomatic leaves at the four sampling time points, with the relative abundances between 56 and 92% (Figure 2A). Before spraying, the dominant genus in asymptomatic leaves was *Alternaria* (33%), followed by *Cladosporium* (13%), *Symmetrospora* (5%), *Boeremia* (5%), and *Golubevia* (4%). *Golubevia* was the dominant genus in asymptomatic leaves on the 5, 10, and 15th days after spraying, with relative abundances of 35–36%. The relative abundance of *Alternaria* in asymptomatic leaves was less than 4% at three sampling time points after spraying. For bacterial communities, the “others” group had a high percentage in most samples. *Pseudomonas*, *Pantoea*, and *Sphingomonas* were the common dominant genera in symptomatic leaves at four sampling time points (Figure 2B). In asymptomatic leaves, *Weissella* (15%) and *Pantoea* (6%) were the dominant genera in CBJ0, *Pseudomonas*, *Pantoea*, *Sphingomonas*, *Erwinia*, *Romboutsia*, and unidentified Enterobacteriaceae were the dominant genera in CBJ1, CBJ2, and CBJ3, but their relative abundant was less than 8%.

**Figure 3:**
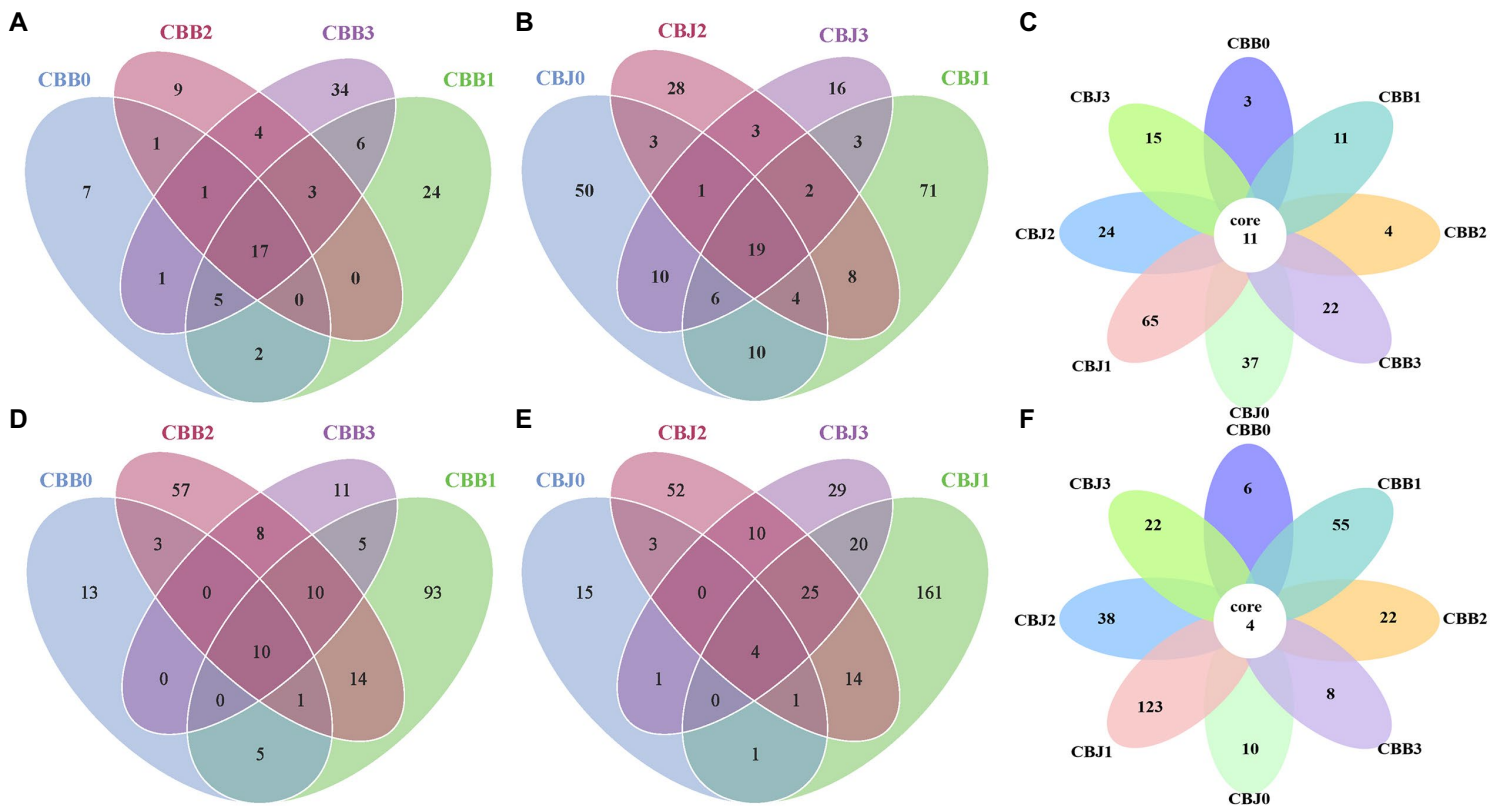
Venn diagram showing the number of microbial OTU detached in tobacco leaves (symptomatic leaves CBB; asymptomatic leaves CBJ) at four sampling time points (0–3). **(A–C)** for fungal, **(D–F)** for bacterial. Numbers in the non-overlapping region indicate unique OTUs for the single sample; numbers in the overlapping region indicate shared OTUs for multi-samples; and numbers in the core region indicate shared OTUs for each sample.

At the four sampling time points, there were 17, 19, and 11 core fungal OTUs in the symptomatic leaves, asymptomatic leaves, and all leaves, respectively (Figures 3A–C). Among the 11 core fungal OTUs, five OTUs belonged to unidentified fungi, while others belonged to *Alternaria*, *Cladosporium*, *Symmetrosporaceae*, *Stagonosporopsis*, *Golubevia*, and *Didymella*. For bacterial communities, there were 10, four, and four core bacterial OTUs in the symptomatic leaves, asymptomatic leaves, and all leaves at four sampling time points, respectively (Figures 3D–F). Among the four core OTUs in all leaves, three OTUs belonged to unidentified bacteria, and the remaining one belonged to *Pantoea*.

### Microbial community diversity

#### Alpha diversity

**Figure 4:**
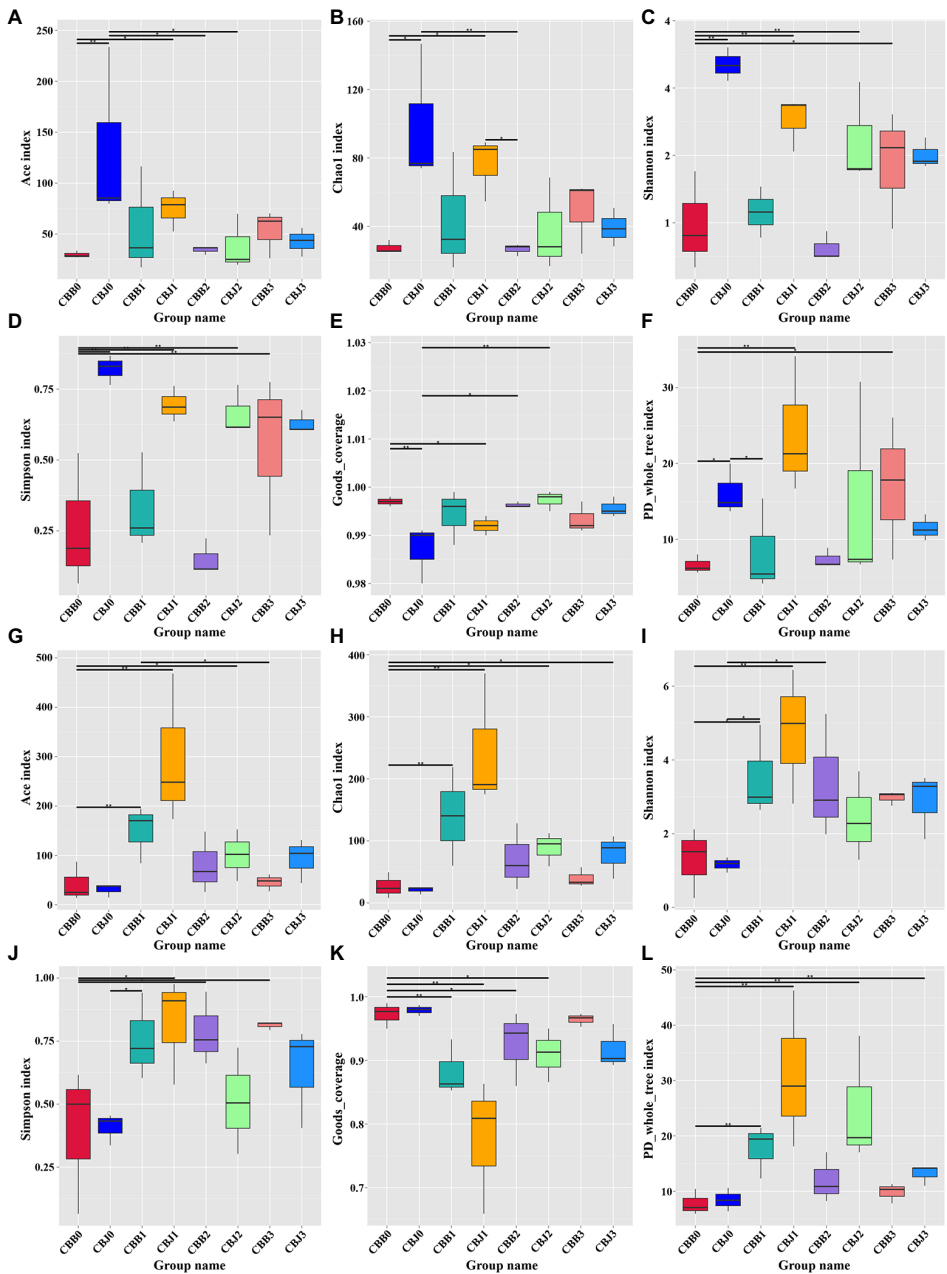
Alpha diversity of the microbial (**A–F** for fungal, **G–L** for bacterial) communities in tobacco leaves (symptomatic leaves CBB; asymptomatic leaves CBJ) at four sampling time points (0–3). **(A,G)** Show the Ace index; **(B,H)** show the Chao1 index; **(C,I)** show the Shannon index; **(D,J)** show the Simpson index; **(E,K)** show the Goods_coverage; and **(F,L)** show the PD_whole_tree index. “*” represents a statistical difference between groups (*p* < 0.05), “**” represents a significant statistical difference between groups (*p* < 0.01; Wilcoxon Signed Rank Test).

Alpha diversity of fungal communities increased in symptomatic leaves and decreased in asymptomatic leaves after STROBY spraying (Figures 4A–F). Before spraying, the Ace index, Chao1 index, Shannon index, Simpson index, and PD_whole_tree index of fungal communities in symptomatic leaves were significantly higher than asymptomatic leaves. At 15 days after spraying, these indices of fungal community in symptomatic leaves seemed lower than asymptomatic leaves, but without statistically significant differences between the two types of leaves. Within 5 days after spraying, the Ace index, Chao1 index, Shannon index, and PD_whole_tree index of the bacterial communities increased significantly in symptomatic leaves (Figures 4G–L).

#### Beta diversity

**Figure 5:**
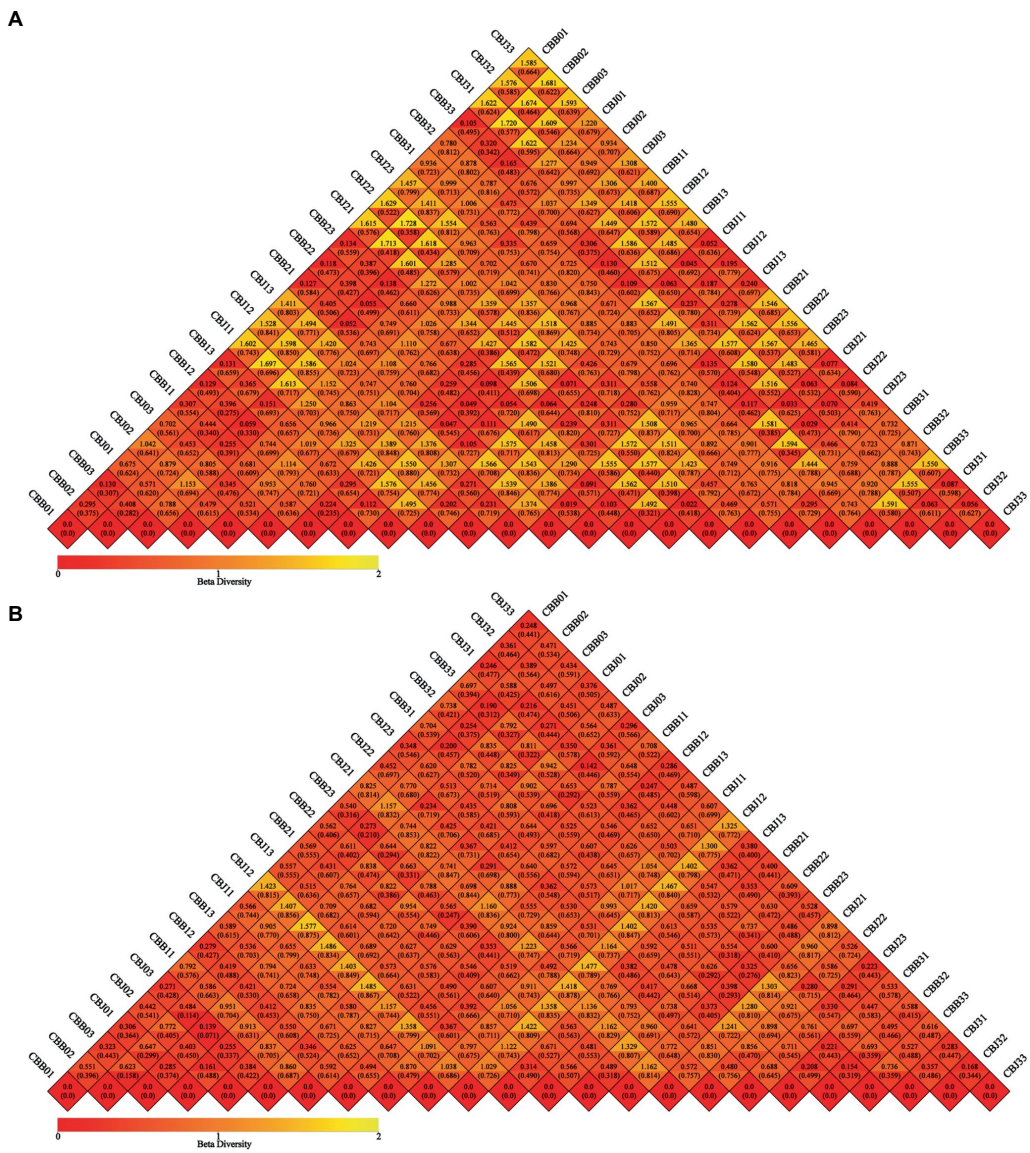
Beta diversity heatmap diagram of microbial communities in tobacco leaves (symptomatic leaves CBB; asymptomatic leaves CBJ) at four sampling time points (The first numbers 0–3 in the sample name represent four different sampling time points, respectively. The numbers 1–3 at the end of the sample name represent three biological replicates.). **(A)** For fungal communities, **(B)** for bacterial communities. In the same grid, the upper and lower values represented Weighted Unifrac and Unweighted Unifrac distance value, respectively.

Weighted and unweighted UniFrac distance metrics were used to estimate microbial beta diversity in the leaf phyllosphere. Dissimilarity coefficients were calculated for all samples and the results confirmed that STROBY could directly affect microbial diversity (Figure 5). Smaller dissimilarity coefficients between samples indicate smaller differences in microbial diversity. Differences between bacterial communities of the different samples were smaller than those of the fungal community. For fungal communities, the CBB0, CBB1, CBB2, and CBB33 differed significantly from CBJ1, CBJ2, and CBJ3. For bacterial communities, only CBJ12 differed from all other samples.

**Figure 6:**
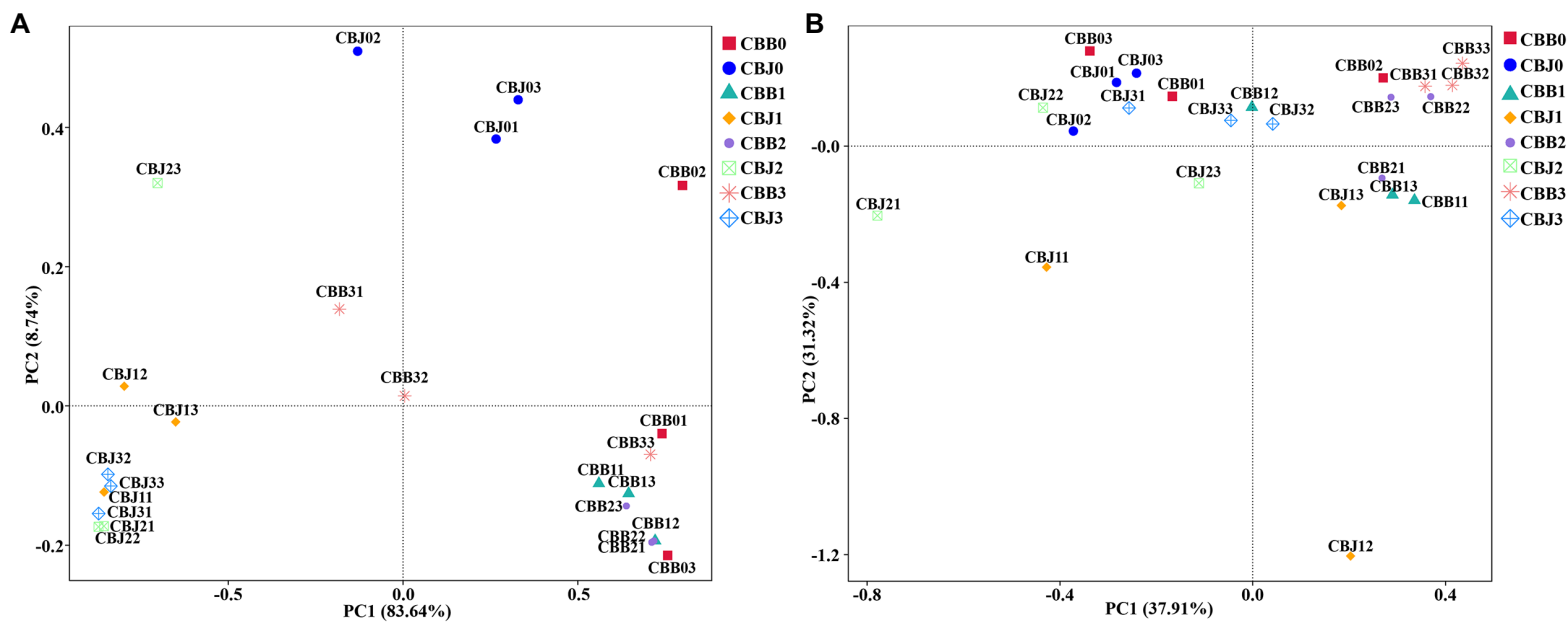
Principal coordinate analysis (PCoA) of the microbial communities in tobacco leaves (symptomatic leaves CBB; asymptomatic leaves CBJ) at four sampling time points (The first numbers 0–3 in the sample name represent four different sampling time points, respectively. The numbers 1–3 at the end of the sample name represent three biological replicates.). **(A)** For fungal communities and **(B)** for bacterial communities.

Principal coordinate analysis (PCoA) of Bray-Curtis dissimilarities was performed to explore how microbial community composition varied after spraying. The fungal communities of symptomatic and asymptomatic leaves were clearly clustered separately (Figure 6A). Most symptomatic samples were clustered in first and fourth quadrants (only CBB31 clustered in second quadrant), and most asymptomatic samples were clustered in second and third quadrants (CBJ01 and CBJ03 clustered in first quadrant), indicating significant differences in fungal community composition between symptomatic and asymptomatic leaves. With the exception of CBB02, CBB31, and CBB32, symptomatic leaves were tightly clustered, showing that no significant changes in the fungal communities of symptomatic leaves until the 15th day after spraying. The group of CBJ0 did not cluster with asymptomatic samples from other sampling time points, indicating that spraying could significantly affect the fungal community composition of asymptomatic leaves. For bacterial communities, most symptomatic samples were clustered in first and fourth quadrants (CBB01 and CBB03 clustered in second quadrant), and most asymptomatic samples were clustered in second and third quadrants (CBJ32 clustered in first quadrant, CBJ12 and CBJ13 clustered in fourth quadrant; Figure 6B). The close sample distance between samples from different sampling time points of the same type of leaves suggested that STROBY application may have little effect on the phyllosphere bacterial communities.

**Figure 7:**
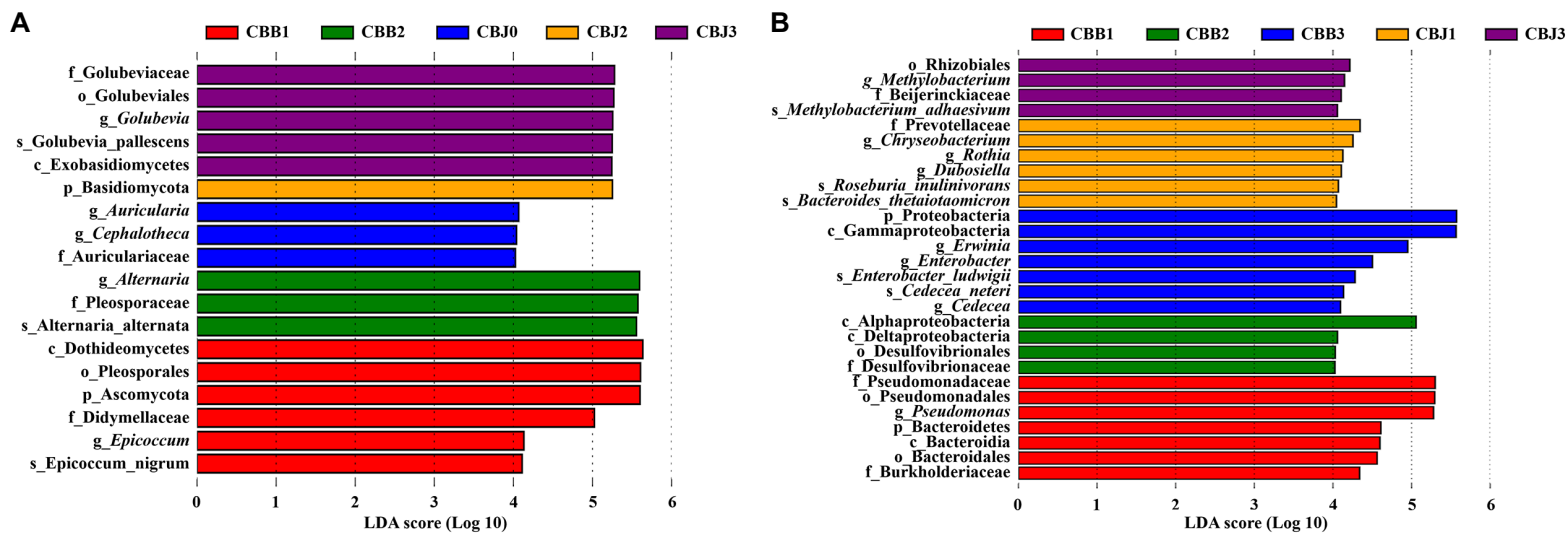
LEfSe analysis of microbial communities among tobacco leaves (symptomatic leaves CBB; asymptomatic leaves CBJ) at four sampling time points (0–3). **(A)** For fungal communities and **(B)** for bacterial communities.

To determine the biomarker in samples, LEfSe was performed to identify taxa that exhibited significant differences among the symptomatic and asymptomatic leaves at different sampling time points (Figure 7). Among the fungal communities, the number of fungal biomarkers was five, one, three, three, and six in CBB1, CBB2, CHJ0, CBJ2, and CBJ3, respectively. In the bacterial communities, a total of 28 bacterial taxa were identified as biomarkers at different sampling points of two types of leaves. The number of bacterial biomarkers in CBB1, CBB2, CBB3, CBJ1, and CBJ3 was four, six, seven, four, and seven, respectively. There were no significantly different fungal biomarkers in CBB0, CBJ1, and CBB3 groups, and no bacterial biomarkers in CBB0, CBJ0, and CBJ2 groups.

#### Microbial community ecological functions

**Figure 8:**
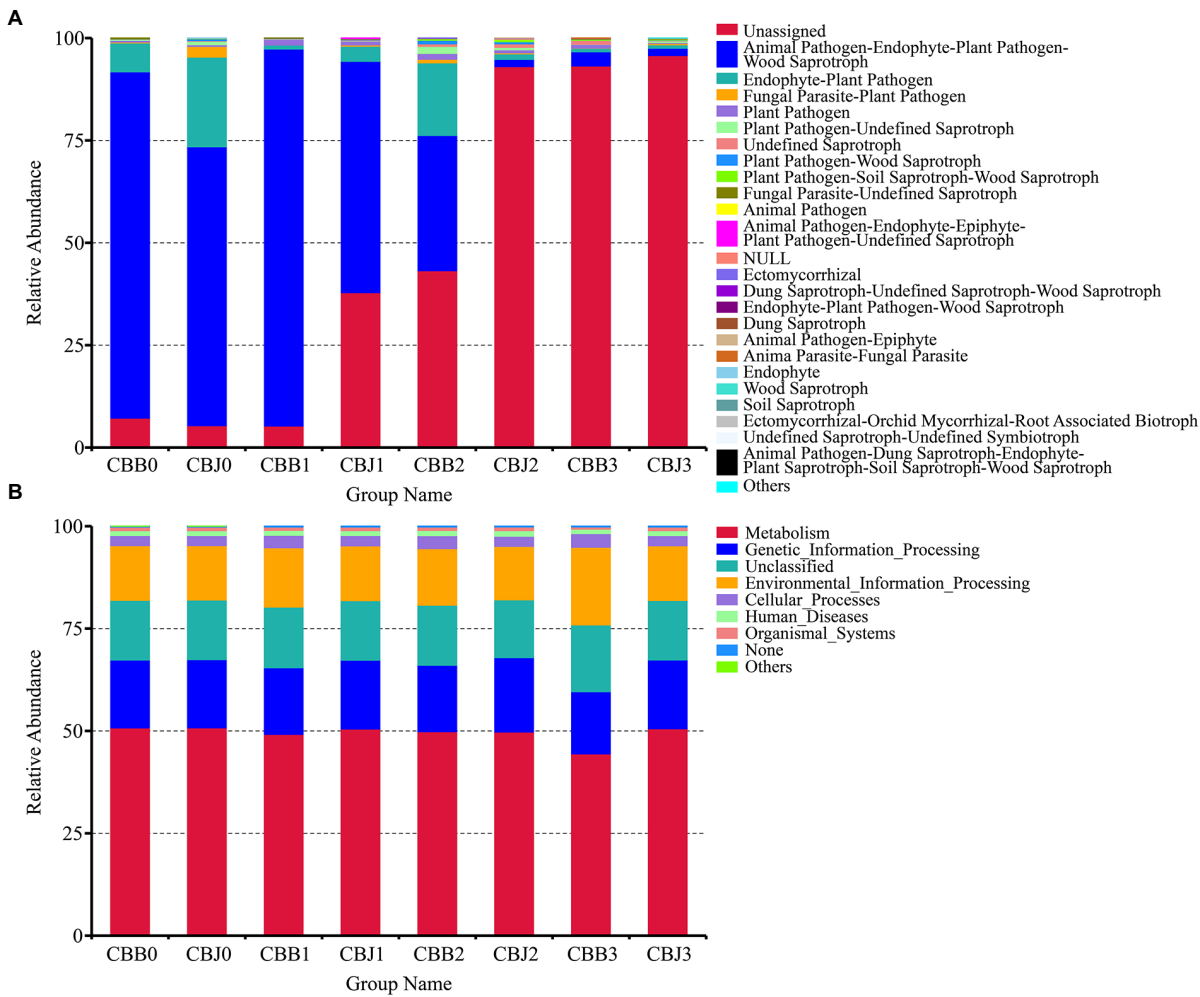
Microbial functional categories of tobacco leaves (symptomatic leaves CBB; asymptomatic leaves CBJ) at four sampling time points (0–3). **(A)** For fungal communities and **(B)** for bacterial communities.

Fungal ecological functions were predicted using FUNGuild based on community composition (Figure 8A). The dominant fungal functions in symptomatic leaves were animal pathogen, endophyte, plant pathogen, wood saprotroph, and unassigned. For fungal communities in CBJ0 group, the dominant functions were unassigned, followed as animal pathogen, endophyte, plant pathogen, and wood saprotroph. At three sampling time points after spraying, the dominant fungal function in asymptomatic leaves was “unassigned,” with relative abundance greater than 95%. Bacterial or archaeal gene families were estimated using PICRUSt (Figure 8B). In this study, metabolism, genetic information processing, and unclassified and environmental information processing were the dominant bacterial community functions common to all samples, only differing in their relative abundance from sample to sample.

### Co-occurrence network analysis between microorganisms

**Figure 9:**
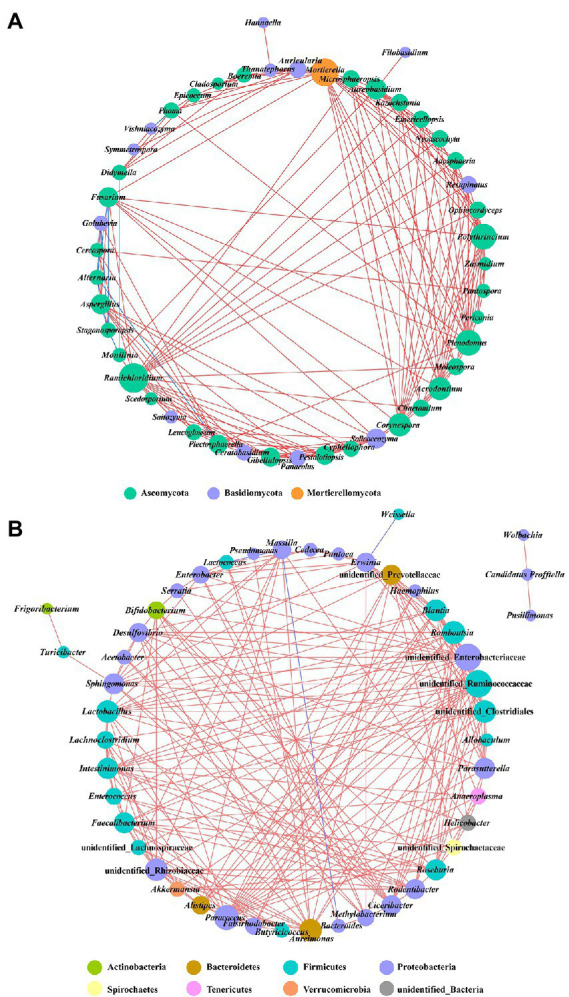
Co-occurrence networks of microbial communities at genus level. **(A)** For fungal communities and **(B)** for bacterial communities. Node color represents the phylum of the genus and the size corresponds to the centrality score. Red lines indicate positive relationships, and blue lines indicate negative relationships.

Co-occurrence network analysis was used to investigate the interaction of complex microorganisms. A total of 10 strong negative correlations and 154 strong positive correlations were identified from 47 fungal genera (Figure 9A). The co-occurring genera were distributed in Ascomycota (72%), Basidiomycota (26%), and Mortierellomycota (2%). As a genus containing the tobacco brown spot pathogen, *Alternaria* was positively correlated with *Stagonosporopsis*, and negatively correlated with *Golubevia*, *Aspergillus*, *Plectosphaerella*, and *Fusarium*. The remaining six of the 10 strong negative correlations of fungal communities were *Golubevia*-*Stagonosporopsis*, *Didymella*-*Monilinia*, *Aspergillus*-*Stagonosporopsis*, *Stagonosporopsis*-*Fusarium*, *Stagonosporopsis*-*Monilinia*, and *Phoma*-*Vishniacozyma*. *Ramichloridium*, *Mortierella*, *Plenodomus*, and *Polythrincium* were the top four genera based on the high centrality score. In the bacterial communities, four negative correlations and 212 positive correlations were identified from 48 genera that belong to Proteobacteria (48%), Firmicutes (33%), Bacteroidetes (6%), Actinobacteria (4%), Sporochaetes (2%), Tenericutes (2%), Verrucomicrobia (2%), and unidentified bacteria (2%) (Figure 9B). Four significant negative correlations of bacterial communities occurred in *Erwinia*-*Weissella*, *Bacteroides*-*Aureimonas*, *Lactococcus*-*Massilia*, and *Massilia*-*Aureimonas*. Unidentified Enterobacteriaceae, unidentified Ruminococcaceae, *Paracoccus*, and unidentified Clostridiales were the top four bacterial genera based on the high centrality score.

### The relationship between microorganisms and environmental conditions

**Figure 10:**
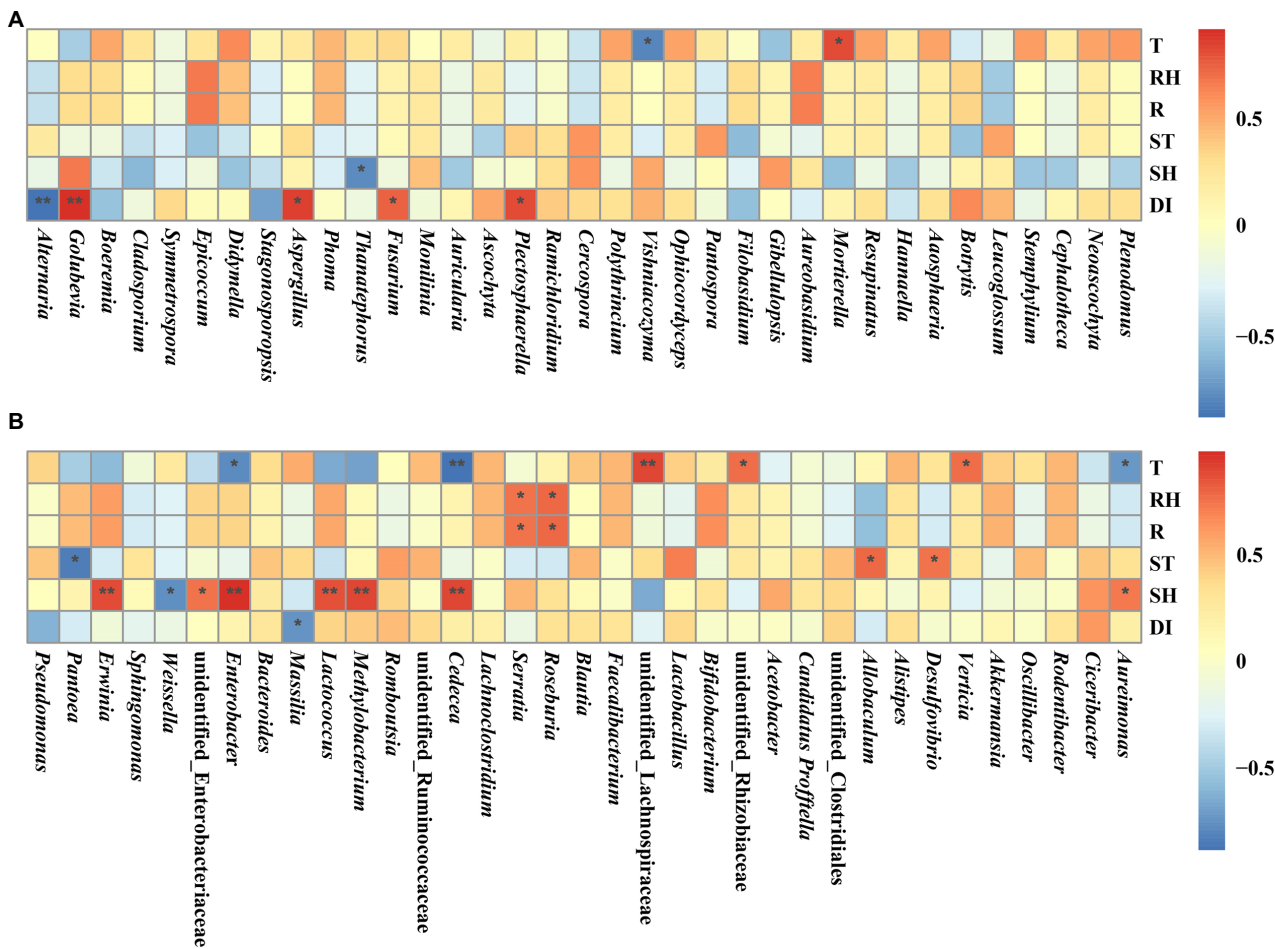
Spearman correlation analysis between microbial communities and environmental factors in tobacco leaves at four sampling time points. **(A)** For fungal communities and **(B)** for bacterial communities. Temperature (T), relative humidity (RH), rainfall (R), soil temperature (ST), soil relative humidity (SH), and disease index (DI). “*” represents a statistical difference between groups (*p* < 0.05), “**” represents a significant statistical difference between groups (*p* < 0.01).

The relationships between microorganisms and temperature, relative humidity, rainfall, soil temperature, soil relative humidity, and disease index were investigated using Spearman’s rank analysis (Figure 10). Temperature was significantly and positively correlated with *Mortierella*, *Verticia*, unidentified Rhizobiaceae, and unidentified Lachnospiraceae, while significantly and negatively correlated with *Vishniacozyma*, *Aureimonas*, *Cedecea*, and *Enterobacter*. Relative humidity and rainfall were significantly and positively correlated with *Roseburia* and *Serratia*. Soil temperature was significantly and positively correlated with *Desulfovibrio* and *Allobaculum*, while significantly and negatively correlated with *Pantoea*. Soil relative humidity was significantly and positively correlated with *Aureimonas*, *Cedecea*, *Methylobacterium*, *Lactococcus*, *Enterobacter*, unidentified Enterobacteriaceae, and *Erwinia*, while significantly and negatively correlated with *Thanatephorus* and *Weissella*. Disease index was significantly and positively correlated with *Plectosphaerella*, *Fusarium*, *Aspergillus*, and *Golubevia*, while significantly and negatively correlated with *Alternaria* and *Massilia*.

## Discussion

A single application of STROBY (300 g/ha, 3000x dilution) in mature tobacco fields could prevent the occurrence of tobacco brown spot on asymptomatic plants, but could not prevent the increase of disease index on symptomatic plants (The disease index increased by 12.2 in 15 days). Perhaps if the rate and dose of STROBY are increased within safe limits, its curative effects on tobacco brown spot may be improved.

As expected, the dominant genus in symptomatic leaves was *Alternaria*, because *Alternatia alternata* is the causal agent of tobacco brown spot. However, we also found a high relative abundance of *Alternaria* in asymptomatic leaves. *Alternaria* on asymptomatic leaves may originate from symptomatic leaves and may cause disease on asymptomatic leaves once environmental conditions are suitable. The relative abundance of *Alternaria* in asymptomatic leaves was lower than symptomatic leaves before spraying. After spraying, the relative abundance of *Alternaria* showed minor fluctuations among symptomatic leaves, and decreased significantly in asymptomatic leaves. In asymptomatic leaves, spores of *Alternaria* may have landed on the leaf surface, or germinated hyphae may have begun initial invasion, but the STROBY application might have killed them off on asymptomatic leaves or halted further progression. On the contrary, in symptomatic leaves, *Alternaria* may had invaded and grown inside the leaf tissue, so that STROBY was unable to eliminate the established growth. We speculate that our conditions of STROBY application (300 g/ha) was able to preventively affect the occurrence of brown spot but did not have strong curative activity. Similar to *Alternaria*, the relative abundance of *Cladosporium* in asymptomatic leaves decreased significantly after spraying. *Cladosporium* contains a number of potential plant pathogens that can cause diseases in a variety of crops such as cucumber, tomato, and canola (Batta, 2004; Rivas and Thomas, 2005; Idnurm et al., 2021). In contrast to *Alternaria*, *Golubevia* in asymptomatic leaves increased significantly after spraying. The co-occurrence network also demonstrated the same result that *Alternaria* and *Golubevia* had a strong negative correlation. We did not find published reports on the effect of *Golubevia* on plant growth, but it can be detected on many plants (Chen et al., 2020; Qu et al., 2020; Liu et al., 2022). In addition to *Alternaria*, the other common fungal OTUs *Cladosporium*, *Didymella* and *Phoma* have been reported to contain tobacco pathogens (Wang et al., 2014; Yuan et al., 2016; Guo et al., 2020).

The leaf phyllosphere bacterial communities did not have as clear a pattern of variation as the fungal community did. Even among the three replicates of the same group, there were large differences in microorganism composition. Overall, the major bacterial genera of the tobacco leaves were *Pseudomonas*, *Pantoea*, *Sphingomonsa*, and *Erwinia*. Within these genera, *Pseudomonas tabaci* (Sinden and Durbin, 1968) and *Pantoea endophytica* (Ilyas et al., 2021) are known as tobacco pathogens.

The alpha diversity results showed that the fungal communities increased in symptomatic leave and decreased in asymptomatic leaves after spraying. We speculated that *Alternaria* as the dominant genus in symptomatic leaves occupied and consumed a large amount of space, nutrients, and free water, resulting in inhibition of the growth of other fungi. When the relative abundance of *Alternaria* decreases, other fungi can grow rapidly, which ultimately lead to an increase in fungal community diversity. Another possible reason was that the fungal community diversity in asymptomatic leaves was significantly higher than symptomatic leaves before spraying. Due to defense mechanisms of plants, many fungi on asymptomatic leaves may not invade the tissues, but only adhered to the leaf surface, so STROBY had a higher effect on them than the fungi in symptomatic leaves. And thereby, the fungal community diversity of asymptomatic leaves decreased after spraying. Additionally, the diversity of bacterial community increased in all leaves after spraying. On the one hand, the STROBY action targets mainly fungal communities such as Ascomycetes, Basidiomycetes, Deuteromycetes, and Oomycetes (Grossmann and Retzlaff, 1997). On the other hand, the resources on tobacco leaves are limited, and once the fungal communities are inhibited, the resources they consume will also be reduced, so more resource will be available for use by bacterial communities.

By using community dissimilarity analysis between consecutive sampling time points, the community change trends were visualized. The change rate of fungal communities was fastest within 5 days after spraying in asymptomatic leaves, and at 10–15 days after spraying in symptomatic leaves. As speculated upon and mentioned above, fungi on symptomatic leaves may invade the inside of leaves through necrotic spot tissue. Since the structural integrity of asymptomatic leaves makes it difficult for fungi to invade the interior, the majority of fungi may only adhere to the leaf surface. Therefore, on asymptomatic leaves, STROBY can directly affect the fungal communities, while on symptomatic leaves, STROBY needs to be absorbed by leaves to affect the fungal communities. These may account for the delayed action of STROBY on fungal communities of symptomatic leaves compared to those of asymptomatic leaves. For the same reasons, the fungal communities of the asymptomatic leaves varied to a greater extent than symptomatic leaves throughout the experiment. This observation is supported by the shift in the function of the phyllosphere microbial community. The relative abundance of plant pathogen, endophyte, and wood saprotroph decreased significantly with 5 days after spraying in asymptomatic leaves and 10–15 days after spraying in symptomatic leaves. Ultimately unassigned fungal communities became dominant and occupied the vast majority of phyllosphere niche. However, the function of phyllosphere bacterial communities did not fluctuate significantly throughout the experiment. This further confirms that STROBY may not have strong direct effects on bacteria.

The co-occurrence network demonstrated the relationship of phyllosphere microbial communities, which may provide crucial details on the interactions (such as parasitism, competition, symbiosis, and mutualism) that exist between different populations (Deng et al., 2012; Vacher et al., 2016). In this study, the node size of the co-occurrence network of the phyllosphere bacterial community was higher than that of the phyllosphere fungal community, indicating that the interactions between phyllosphere bacteria were stronger than between fungi. Among the interactions between microorganisms, the majority was positive interactions, showing directly correlated increases or decreases in population, which meant that the most phyllosphere microorganisms were affected the same way by external pressures, and speculatively may be more symbiotic or mutualistic rather than competitive, or did not exert major effects on each other.

## Conclusion

This study demonstrated variations in the microbial community of the leaf phyllosphere with and without tobacco brown spot before and after STROBY application. After STROBY application, visible disease did not appear on asymptomatic leaves and the disease index of symptomatic plants disease not increase greatly. *Alternaria* was found to be detectable even on asymptomatic leaves in tobacco fields with greater than 25% incidence of brown spot. In addition to *Alternaria*, fungal genera known to contain plant pathogens such as *Cladosporium*, *Didymella*, *Phoma*, *Pseudomonas*, and *Pantoea* were also found to be present on tobacco leaves. After spraying, the fungal community diversity was significantly reduced in symptomatic leaves. The bacterial community diversity did not change significantly. The effect of STROBY on the structure of leaf phyllosphere fungal communities was significantly higher than bacterial communities, and had the greatest effect on *Alternaria*.

## Data availability statement

The datasets presented in this study can be found in online repositories. The names of the repository/repositories and accession number(s) can be found at: https://www.ncbi.nlm.nih.gov/, PRJNA806570.

## Author contributions

H-CW and Z-HY contributed to conception. L-GX, L-TC, TG, and FL conducted the study, organized the database, performed the statistical analysis, and wrote the first draft of the manuscript. H-CW and TH revised the manuscript and wrote some sections. All authors contributed to the article and approved the submitted version.

## Funding

This research was financed by China National Tobacco Corporation [110202101048(LS-08) and 110202001035(LS-04)], the “Hundred” Level Innovative Talent Foundation of Guizhou Province (GCC[2022]028-1), National Natural Science Foundation of China (31960550 and 32160522), Guizhou Science Technology Foundation (ZK[2021]Key036), Guizhou Province Technology R&D Program (Grant number: [2019]2398), and Guizhou Tobacco Company (2020XM03 and 2020XM22). The authors declare that this study received funding from Guizhou Tobacco Company. The funder was not involved in the study design, collection, analysis, interpretation of data, the writing of this article or the decision to submit it for publication.

## Conflict of interest

The authors declare that the research was conducted in the absence of any commercial or financial relationships that could be construed as a potential conflict of interest.

## Publisher’s note

All claims expressed in this article are solely those of the authors and do not necessarily represent those of their affiliated organizations, or those of the publisher, the editors and the reviewers. Any product that may be evaluated in this article, or claim that may be made by its manufacturer, is not guaranteed or endorsed by the publisher.

## Footnotes

^1^ http://qiime.org/scripts/split_libraries_fastq.html

^2^ http://www.drive5.com/usearch/manual/uchime_algo.html

^3^ http://picrust.github.com/

^4^ https://github.com/UMNFuN/FUNGuild
