# Supplementary material for: Variations in leaf phyllosphere microbial communities and development of tobacco brown spot before and after fungicide application
Source: Front Microbiol. 2022 Nov 17;13:1068158. doi: 10.3389/fmicb.2022.1068158 (PMC9714265; doi:10.3389/fmicb.2022.1068158)
Supplement: Supplementary file 1 [file Table_1.DOCX]

Table 1 Environmental factors and symptomatic tobacco plant disease indices in tobacco fields

| Sampling date | Temperature (^o^C) | Relative humidity (%) | Rainfall (mm/m^2^) | Soil temperature (^o^C) | Soil relative humidity (%) | Disease index |
| --- | --- | --- | --- | --- | --- | --- |
| Aug25 | 19.0 | 62.2 | 0.0 | 21.7 | 27.3 | 17.9 |
| Aug26 | 17.6 | 65.5 | 0.0 | 21.1 | 24.9 | 18.0 |
| Aug27 | 22.2 | 62.7 | 0.0 | 21.7 | 22.6 | 18.0 |
| Aug28 | 19.2 | 82.4 | 1.4 | 21.6 | 21.5 | 18.2 |
| Aug29 | 18.3 | 82.5 | 13.6 | 21.1 | 23.5 | 18.3 |
| Average | 19.3+1.8a | 71.1+10.5b | 3.0+6.0a | 21.4+0.3a | 24.0+2.2b | 18.1+0.2c |
| Aug30 | 19.1 | 80.4 | 0.6 | 21.2 | 25.2 | 20.0 |
| Aug31 | 19.8 | 77.8 | 3.0 | 21.3 | 24.8 | 20.0 |
| Sep1 | 19.6 | 73.4 | 0.0 | 21.5 | 23.7 | 20.6 |
| Sep2 | 19.9 | 77.3 | 0.0 | 21.5 | 22.3 | 20.6 |
| Sep3 | 19.0 | 83.8 | 19.0 | 21.2 | 27.7 | 20.8 |
| Average | 19.5+0.4a | 78.5+3.9ab | 4.5+8.2a | 21.3+0.2a | 24.7+2.0b | 20.4+0.4c |
| Sep4 | 19.8 | 81.2 | 1.2 | 21.5 | 30.2 | 21.6 |
| Sep5 | 20.2 | 81.9 | 2.2 | 21.8 | 28.6 | 23.2 |
| Sep6 | 16.1 | 89.5 | 53.6 | 20.1 | 33.2 | 25.7 |
| Sep7 | 17.5 | 74.8 | 0.0 | 21.0 | 32.1 | 27.4 |
| Sep8 | 17.5 | 79.0 | 0.0 | 21.4 | 31.1 | 28.3 |
| Average | 18.2+1.7a | 81.3+5.4ab | 11.4+23.6a | 21.2+0.7a | 31.0+1.8a | 25.2+2.8b |
| Sep9 | 18.9 | 79.1 | 0.0 | 21.9 | 30.6 | 28.5 |
| Sep10 | 17.4 | 89.8 | 11.8 | 20.8 | 32.5 | 29.0 |
| Sep11 | 16.1 | 86.5 | 0.0 | 20.2 | 32.5 | 30.0 |
| Sep12 | 16.8 | 90.8 | 13.0 | 20.2 | 32.9 | 31.6 |
| Sep13 | 16.5 | 91.0 | 25.4 | 19.9 | 34.4 | 32.2 |
| Average | 17.1+1.1a | 87.4+5.0a | 10.0+10.6a | 20.6+0.8a | 32.6+1.4a | 30.3+1.6a |

Different letters in the same column indicate significant difference at *P* < 0.05 among stage

Table S1 Statistic information on the metagenetic sequence of different tobacco leaf samples

| Sequence  type | Sample Name^1^ | Raw reads (#) | Clean reads (#) | Base (nt) | Average length (nt) | Q20 | Q30 | GC % |
| --- | --- | --- | --- | --- | --- | --- | --- | --- |
| Bacteria | CBB01 | 90,748 | 67,483 | 27,404,411 | 406 | 98.75 | 95.58 | 55.61 |
| Bacteria | CBB02 | 94,573 | 64,865 | 26,396,105 | 407 | 98.83 | 95.71 | 55.7 |
| Bacteria | CBB03 | 90,962 | 65,452 | 26,571,559 | 406 | 98.79 | 95.66 | 55.69 |
| Bacteria | CBB11 | 105,525 | 66,785 | 26,978,905 | 404 | 98.52 | 95.17 | 53.37 |
| Bacteria | CBB12 | 116,164 | 69,526 | 28,263,606 | 407 | 97.9 | 93.51 | 55.57 |
| Bacteria | CBB13 | 98,705 | 62,369 | 25,475,581 | 408 | 98.67 | 95.44 | 54.91 |
| Bacteria | CBB21 | 108,464 | 67,112 | 27,304,950 | 407 | 98.8 | 95.82 | 55.55 |
| Bacteria | CBB22 | 104,446 | 66,937 | 27,446,755 | 410 | 98.89 | 96.04 | 54.06 |
| Bacteria | CBB23 | 114,037 | 67,953 | 28,208,772 | 415 | 98.28 | 94.44 | 54.38 |
| Bacteria | CBB31 | 112,238 | 68,217 | 28,563,684 | 419 | 96.91 | 91.12 | 55.13 |
| Bacteria | CBB32 | 106,101 | 67,065 | 28,426,178 | 424 | 98.68 | 95.4 | 54.67 |
| Bacteria | CBB33 | 95,570 | 60,969 | 26,044,026 | 427 | 98.74 | 95.63 | 55.29 |
| Bacteria | CBJ01 | 89,129 | 63,356 | 25,720,821 | 406 | 98.77 | 95.67 | 55.69 |
| Bacteria | CBJ02 | 95,326 | 64,998 | 26,320,634 | 405 | 98.82 | 95.75 | 55.56 |
| Bacteria | CBJ03 | 80,971 | 67,842 | 27,555,274 | 406 | 98.74 | 95.53 | 55.69 |
| Bacteria | CBJ11 | 105,076 | 67,152 | 27,072,647 | 403 | 98.58 | 95.24 | 55.44 |
| Bacteria | CBJ12 | 112,332 | 68,308 | 26,364,768 | 386 | 98.58 | 95.32 | 54.74 |
| Bacteria | CBJ13 | 100,300 | 67,481 | 27,398,619 | 406 | 98.7 | 95.52 | 55.42 |
| Bacteria | CBJ21 | 112,694 | 69,602 | 28,244,564 | 406 | 97.61 | 92.96 | 55.19 |
| Bacteria | CBJ22 | 108,678 | 64,225 | 26,053,765 | 406 | 97.62 | 92.97 | 55.13 |
| Bacteria | CBJ23 | 99,413 | 64,441 | 26,147,162 | 406 | 98.71 | 95.59 | 55.44 |
| Bacteria | CBJ31 | 103,488 | 63,322 | 25,702,661 | 406 | 98.84 | 95.91 | 55.66 |
| Bacteria | CBJ32 | 115,298 | 69,267 | 28,175,412 | 407 | 98.79 | 95.79 | 55.62 |
| Bacteria | CBJ33 | 96,467 | 60,497 | 24,593,935 | 407 | 98.85 | 95.92 | 55.6 |
| Fungi | CBB01 | 83,528 | 81,656 | 18,362,030 | 225 | 98.89 | 97.16 | 44.5 |
| Fungi | CBB02 | 91,889 | 89,611 | 20,038,268 | 224 | 98.6 | 96.82 | 45.96 |
| Fungi | CBB03 | 83,205 | 80,893 | 18,403,402 | 228 | 98.36 | 96.25 | 45.27 |
| Fungi | CBB11 | 108,191 | 64,043 | 14,603,109 | 228 | 95.68 | 92.27 | 50.64 |
| Fungi | CBB12 | 100,935 | 68,340 | 16,314,280 | 239 | 95.3 | 91.45 | 51.17 |
| Fungi | CBB13 | 105,158 | 67,519 | 16,846,284 | 250 | 93.52 | 88.68 | 54.07 |
| Fungi | CBB21 | 111,494 | 66,188 | 16,302,774 | 246 | 94.14 | 89.65 | 52.7 |
| Fungi | CBB22 | 100,043 | 60,848 | 14,811,026 | 243 | 94.7 | 90.64 | 51.97 |
| Fungi | CBB23 | 109,541 | 65,397 | 16,789,522 | 257 | 91.72 | 85.9 | 56.54 |
| Fungi | CBB31 | 102,695 | 65,403 | 17,790,525 | 272 | 89.17 | 81.85 | 60.7 |
| Fungi | CBB32 | 100,789 | 62,777 | 16,571,969 | 264 | 89.52 | 82.37 | 60.78 |
| Fungi | CBB33 | 99,145 | 60,614 | 14,337,641 | 237 | 95.67 | 92.14 | 50.38 |
| Fungi | CBJ01 | 89,630 | 70,938 | 18,435,328 | 260 | 92.52 | 86.8 | 55.9 |
| Fungi | CBJ02 | 97,009 | 80,727 | 21,283,772 | 264 | 92.43 | 86.89 | 57.12 |
| Fungi | CBJ03 | 93,007 | 81,523 | 21,350,562 | 262 | 92.63 | 87.01 | 56.72 |
| Fungi | CBJ11 | 108,739 | 65,622 | 17,862,339 | 272 | 89 | 81.61 | 61.36 |
| Fungi | CBJ12 | 107,113 | 68,297 | 18,587,813 | 272 | 88.91 | 81.53 | 61.28 |
| Fungi | CBJ13 | 107,513 | 69,700 | 18,912,486 | 271 | 89.12 | 81.49 | 61.11 |
| Fungi | CBJ21 | 98,403 | 62,259 | 16,810,986 | 270 | 88.5 | 80.85 | 61.28 |
| Fungi | CBJ22 | 112,174 | 68,334 | 18,588,060 | 272 | 88.58 | 81.17 | 61.18 |
| Fungi | CBJ23 | 110,242 | 68,251 | 18,648,285 | 273 | 89.31 | 82.07 | 61.02 |
| Fungi | CBJ31 | 105,236 | 69,891 | 18,923,871 | 271 | 88.72 | 81.18 | 61.41 |
| Fungi | CBJ32 | 111,320 | 66,067 | 17,965,804 | 272 | 88.76 | 81.3 | 61.35 |
| Fungi | CBJ33 | 104,562 | 64,186 | 17,394,381 | 271 | 88.77 | 80.94 | 61.35 |

^1^ CBB = Symptomatic leaves, CBJ = Asymptomatic leaves. The first numbers 0, 1, 2, and 3 in the sample name represent four different sampling time points, respectively. The numbers 1, 2, and 3 at the end of the sample name represent three biological replicates.
